# Demography of the Giant Otter (*Pteronura brasiliensis*) in Manu National Park, South-Eastern Peru: Implications for Conservation

**DOI:** 10.1371/journal.pone.0106202

**Published:** 2014-08-27

**Authors:** Jessica Groenendijk, Frank Hajek, Paul J. Johnson, David W. Macdonald, Jorge Calvimontes, Elke Staib, Christof Schenck

**Affiliations:** 1 San Diego Zoo Global Peru, Department of Cusco, Cusco, Perú; 2 Nature Services Peru, Department of Cusco, Cusco, Perú; 3 Wildlife Conservation Research Unit, University of Oxford, Abingdon, Oxfordshire, United Kingdom; 4 Environmental Studies and Research Center, University of Campinas, São Paulo, Brazil; 5 Frankfurt Zoological Society, Frankfurt, Germany; Cornell University College of Veterinary Medicine, United States of America

## Abstract

The giant otter (*Pteronura brasiliensis*) is an endangered semi-aquatic carnivore of South America. We present findings on the demography of a population inhabiting the floodplain of Manu National Park, south-eastern Peru, arising from 14 annual dry season censuses over a 16 year period. The breeding system of territorial groups, including only a single breeding female with non-reproductive adult ‘helpers’, resulted in a low intrinsic rate of increase (0.03) and a slow recovery from decades of hunting for the pelt trade. This is explained by a combination of factors: (1) physiological traits such as late age at first reproduction and long generation time, (2) a high degree of reproductive skew, (3) small litters produced only once a year, and (4) a 50% mortality between den emergence and age of dispersal, as well as high mortality amongst dispersers (especially males). Female and male giant otters show similar traits with respect to average reproductive life-spans (female 5.4 yrs., male 5.2 yrs.) and average cub productivity (female 6.9, male 6.7 cubs per lifetime); the longest reproductive life spans were 11 and 13 years respectively. Individual reproductive success varied substantially and depended mainly on the duration of dominance tenure in the territory. When breeding females died, the reproductive position in the group was usually occupied by sisters or daughters (n = 11), with immigrant male partners. Male philopatry was not observed. The vulnerability of the Manu giant otter population to anthropogenic disturbance emphasises the importance of effective protection of core lake habitats in particular. Riverine forests are the most endangered ecosystem in the Department of Madre de Dios due to the concentration of gold mining, logging and agricultural activities in floodplains, highlighting the need for a giant otter habitat conservation corridor along the Madre de Dios River.

## Introduction

Long-term population studies yield the vital rates and demographic data necessary to understand the factors responsible for changes in populations and hence allow the evaluation of the effectiveness of conservation management decisions [1], [2]. For large carnivores, that typically occur at low absolute densities, demography is especially relevant for Protected Area design [3] and direct management interventions such as habitat zoning.

The giant otter (*Pteronura brasiliensis,* Zimmermann 1780) is a semi-aquatic carnivore of South America, occurring east of the Andes in the Orinoco, Amazonas, and Parana basins, and in the Guianas [4], [5]. Between the mid 1940s and 1973, when a high demand for giant otter skins led to professional, uncontrolled hunting for the pelt trade, the giant otter was extirpated from much of its southern and easterly range including Uruguay, Paraguay and Argentina, and east of the Tocantins and Parana basins in Brazil [5], [6], [7]. At least 23,162 pelts were officially exported from the Peruvian Amazon during this period [8], with the annual number declining steadily after 1960; the marked reduction in export was likely due to greatly depleted giant otter populations in areas accessible to hunters [9].

In 1973, the commercial hunting of wildlife was banned in Peru and other countries of the giant otter’s range, and the species was listed under Appendix 1 of CITES (Convention of International Trade in Endangered Species) in 1975 [10], effectively leading to the collapse of the international pelt market. Despite this, almost two decades later, in 1993, the species was upgraded from ‘Vulnerable’ to its current ‘Endangered’ IUCN Red List status [11].

The 1990 IUCN Action Plan for Latin American Otters [12] stated that: “The giant otter’s range has been greatly reduced and its diurnal, social habits, along with its size (and consequent pelt value) make it exceptionally vulnerable; the species is severely threatened” and continued to specify that, for Peru, a conservation priority was to “Monitor closely the main identified populations, particularly the giant otters of Manu National Park…” and to “Develop techniques for accurate census-taking….” This study was initiated to respond to these needs.

Thus, the objectives of this paper are to:

1. Present demographic data from the first 16 years of a giant otter research and conservation project conducted in Manu National Park, south-eastern Peru. Baseline demographic and reproductive variables, including survivorship; longevity; average age at dispersal; earliest and average age at primiparity; average litter size, frequency and seasonality; average reproductive lifespan; and variance in reproductive success are documented.
2. Perform a cohort life history analysis with these data in order to parameterise a life table and calculate the net reproductive rate, generation time and intrinsic rate of increase for the Manu population [13].
3. Discuss the implications of our findings for giant otter conservation in Manu National Park and south-eastern Peru.

### Giant otter natural history

Most mustelids live solitarily and few live in groups [14]. The social system of giant otters is therefore unusual [15]; a typical giant otter population consists of highly cohesive multi-male/female families with defended territories, plus transients that have left their natal groups on attaining sexual maturity [4], [16], [17]. Family groups are generally composed of a monogamous breeding pair and their offspring of several years, numbering 2–16 individuals [4], [5], [16], [18]. The dominant pair in each family produces a litter once a year and other adults do not breed [16], [18]. Alloparenting includes the feeding of cubs and teaching of hunting skills by sub-adults and adults, and may also occur in the form of ‘babysitting’ in the den [16], [19]. Observations of wild and captive otters suggest that giant otters are weaned at approx. 6 months old [17], [20] and rely to a great extent on other group members for prey provision until 1.5 years old [16]. Group and territory defence is cooperative [7], [21], though intra-specific agonistic encounters are rarely observed [4], [16], [21] with scent marking at latrines thought to be important for territorial demarcation [4], [16], [18].

Unlike many other otter species, sexual dimorphism in giant otters is not pronounced: adult male total body length ranges between 1.5 to 1.8 m, while females are marginally smaller at 1.5 to 1.7 m. Adult males weigh between 23 and 32 kg and females between 20 and 29 kg [4], [7], [19]. Observations of captive individuals suggest that giant otters of both sexes reach sexual maturity at between 2 and 3 years of age [20], [22].

Giant otters feed almost exclusively on fish [4], [6], [7], [16], [23]. Although group members hunt together, each individual captures and consumes its own prey. An adult giant otter may eat 3–4 kg of food per day [7], [16]. Giant otters hunt opportunistically in non-ideal conditions, (for example, during the high-water period), or more selectively in optimal conditions [16], [24], [25]. Although cooperation between group members during hunting is not fully understood, larger groups (8 inds.) spend significantly less time obtaining food than do smaller groups (5 inds.), and have higher fish capture rates [16].

Reproductive suppression is likely to occur in giant otters, as only the dominant pair in each family breeds [4]. Gestation is between 64 to 77 days [20], [26], [27], [28]. Pseudo-pregnancies are common in captivity [20]; for example, in Cali Zoo, Colombia, the reproductive activity of a pair was monitored for 5 years with the female giving birth to nine litters. In that time, she experienced four pseudo-pregnancies [20], [28], [29]. Pseudo-pregnancies have not been conclusively documented in the wild. Delayed implantation, suggested by Sykes-Gatz [20] for giant otters in captivity, was confirmed by Corredor and Muñoz [29] at Cali Zoo. Reproductive success is greater in territories with large areas of lake, where more young are produced, and are guarded and provisioned by non-breeding adults. In addition, cubs produced in these territories are more likely to disperse successfully (become breeders at least once away from the natal territory) [unpublished].

### Study Area

**Figure 1:**
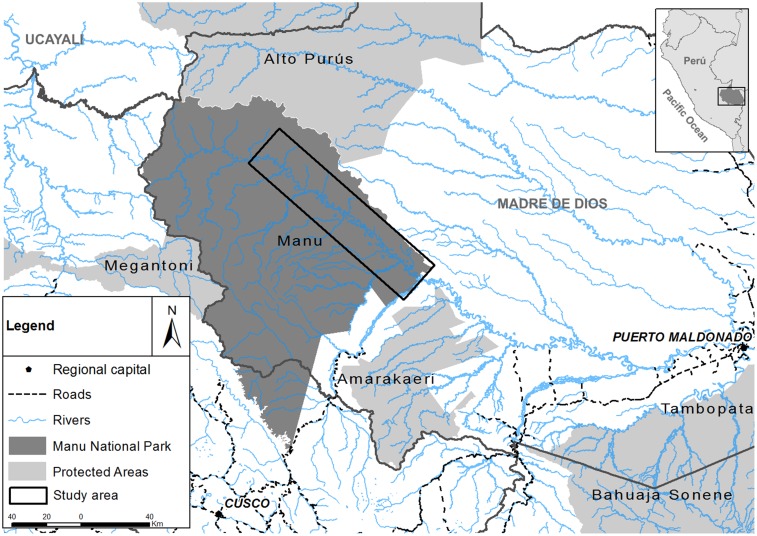
The study area (large black rectangle), Manu National Park, south-eastern Peru.

Manu National Park (16,921 km^2^) lies in the Department of Madre de Dios, south-eastern Peru, at the foothills of the tropical Andes. Created in 1973, it is a World Heritage Site [30] and is located on the border of the Tropical Andes biodiversity hotspot [31]. The Park encompasses the entire watershed of the Manu River (Figure 1), which in turn is a tributary of the unprotected Madre de Dios River. Evergreen tropical forest dominates the Manu River flood plain.

In its lower stretches, the Manu is a lowland, white-water river, varying in width between 150 m and 200 m, with sandy beaches and frequent meanders. River flow varies considerably between the rainy season (November to April) and the dry season (May to October), with pronounced river level fluctuations. As the river current erodes the bank, some meanders are cut off to form oxbow lakes, which can persist as distinct bodies of water for decades or centuries, but which remain connected to the parent river to varying degrees [32]. As the lakes are all formed by the Manu River, lake depth is not highly variable; average lake depth is 2.04 m (range 0.5–4.89 m, SD = 0.97, n = 22) [33]. White-water lakes are nutrient rich and highly productive; they have considerably higher fish biomass per unit area than their associated river channels - one study reports a fish biomass density of 13.4 g m^−2^ in these lakes compared with 3.4 in their associated water channels [34]. In oxbow lakes, water level fluctuations tend to be less marked (1–3 m), though the strength of the annual floods is highly variable [32].

## Results

In total, 294 different individuals were recorded during the study period (1991–2006), of which 30 were immigrants and 185 were cubs, contributing a total of 884 annual dry season observations to the analysis (a mean of 3.01 observations per otter, range 1–16).

### Population trends

**Figure 2:**
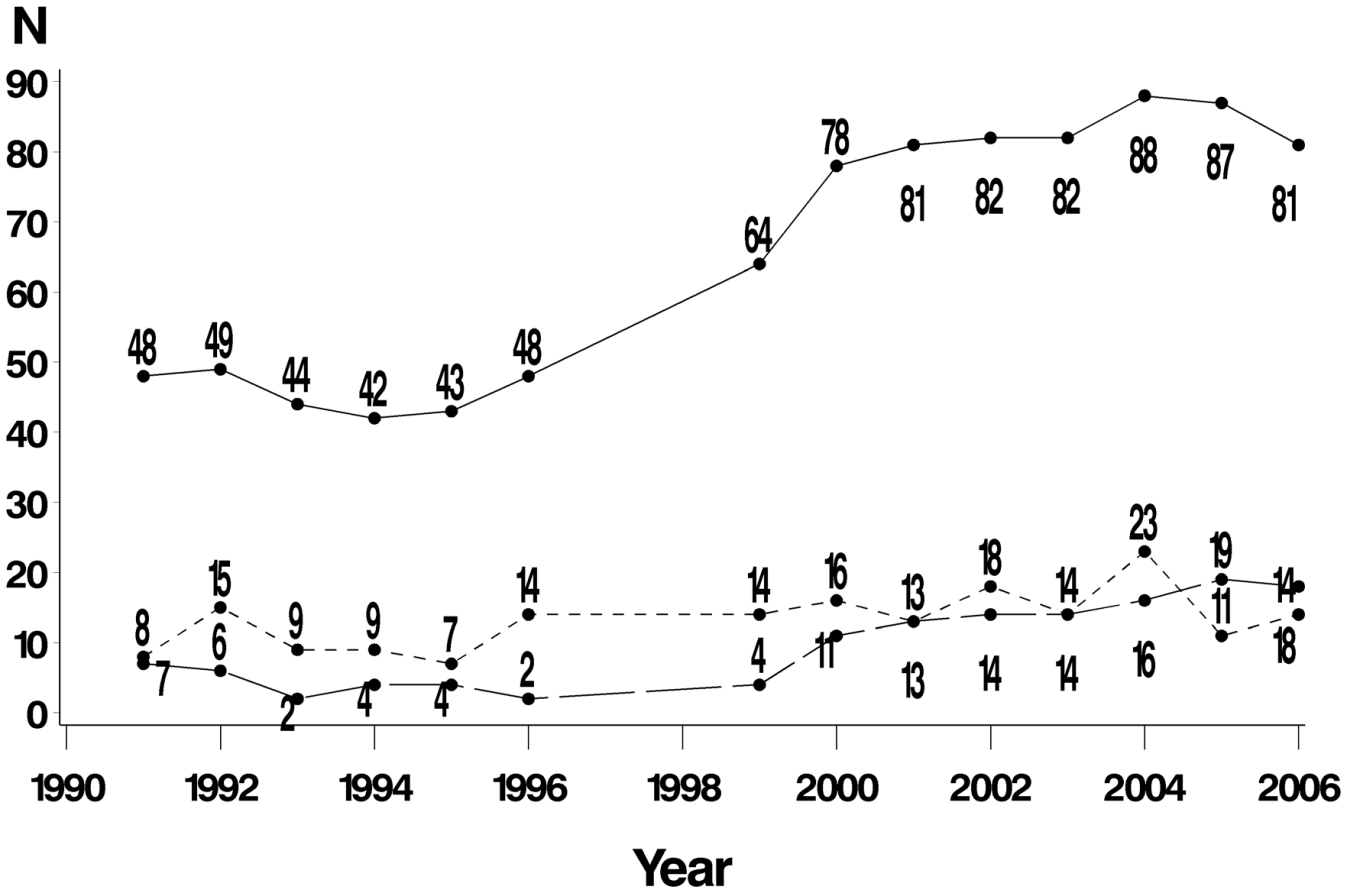
Inferred total number of otters (solid line), cubs (dotted line) and transients (dashed).

**Figure 3:**
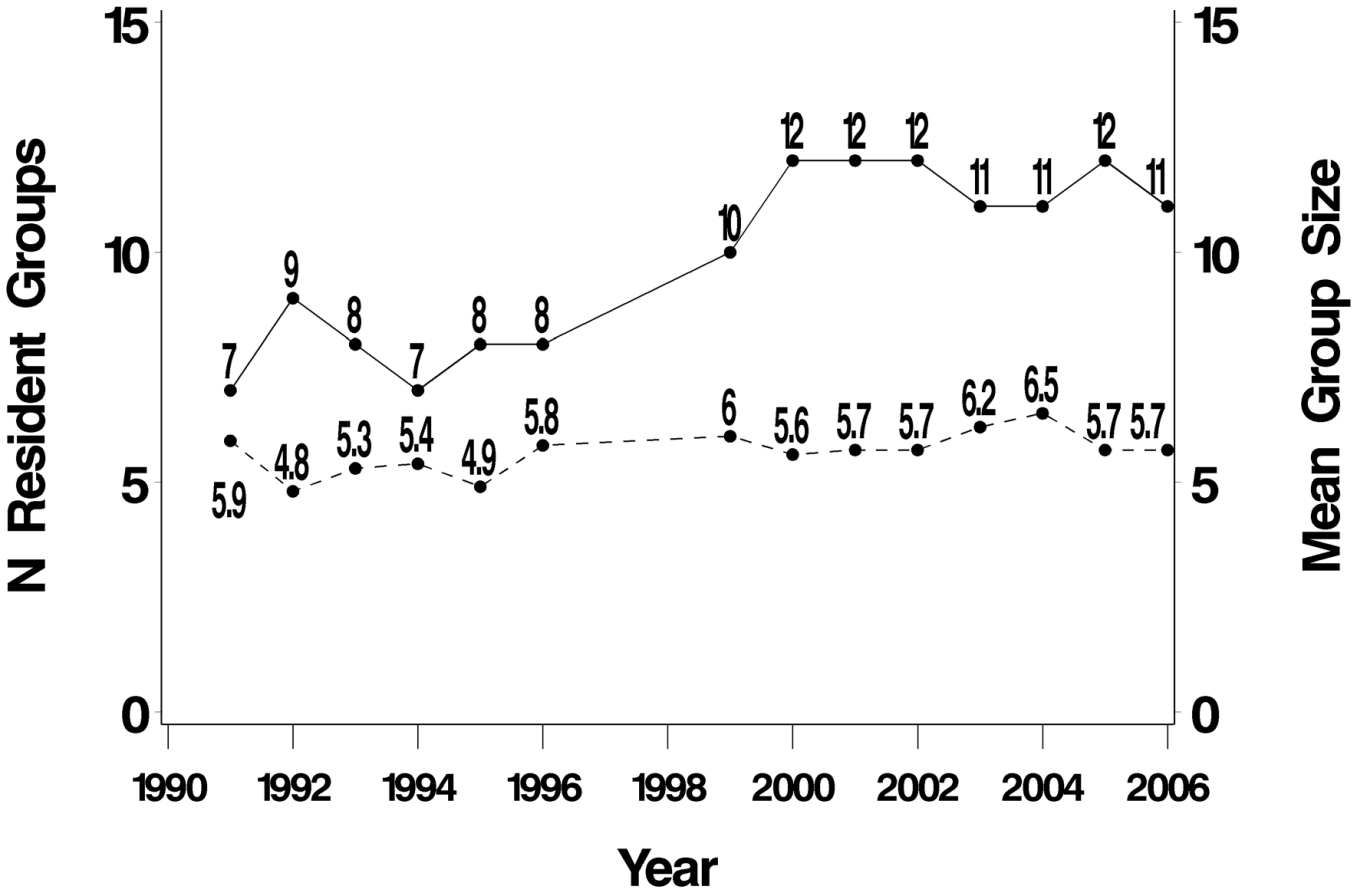
Inferred total number of resident groups (solid line) and mean group size (dotted line).

Observed census totals ranged from 33 animals in 1994 to 80 in 2005 (Table 1, Figure 2). Over the study period, the number of resident giant otter groups increased from a low of 7 in 1991 to a high of 12 in 2005. Observed group size ranged from 2 to 13, with a mean of 6.0 (n = 117). Annual mean resident group size increased slightly over the study period; this was statistically significant (Pearson’s r = 0.56, P = 0.03) (Figure 3).

**Table 1:** Observed census data 1991–2006, Manu National Park, Peru.

	OBSERVED							
Year	Census total	No. res. groups	No. trans.	No. litters	No. cubs	Mean res. group size	Mean litter size	Mean no. cubs/group
1991	43	6	6	3	8	6.2	2.7	1.3
1992	45	8	6	6	13	4.9	2.2	1.6
1993	40	7	1	3	8	5.6	2.7	1.1
1994	33	5	2	4	8	6.2	2.0	1.6
1995	41	8	2	3	7	4.9	2.3	0.9
1996	46	8	0	4	11	5.8	2.8	1.4
1999	53	8	3	6	14	6.3	2.3	1.8
2000	64	10	6	7	16	5.8	2.3	1.6
2001	61	9	5	7	13	6.2	1.9	1.4
2002	63	9	10	7	15	5.9	2.1	1.7
2003	69	9	9	8	14	6.7	1.8	1.6
2004	75	9	10	8	21	7.2	2.6	2.3
2005	80	11	15	6	10	5.9	1.7	0.9
2006	69	10	9	6	14	6.0	2.3	1.4
TOTAL	782	117	84	78	172	6.0	2.2	1.5

A giant otter population is typically described as consisting of family groups and of lone dispersers of both sexes. However, this study confirmed the existence of *transient groups*, fluid single- or mixed-sex associations of multiple non-breeding otters (up to 5 observed) that have not yet secured a territory and do not exhibit strong site fidelity. Lone and group transients made up 13% (range 4% to 22%) of the censused population, with the total number increasing over the study period (Pearson’s r = 0.87, P<0.001). Transient group size ranged from 2 to 5, with a mean of 2.9 (n = 16, se = 0.22).

The observability of resident and transient otters varied across censuses. The mean annual observability of resident groups was 85% (range 71–100%), while that of transients was 59% (range 0–100%). Inferred census totals ranged from 42 individuals in 1994 to 88 individuals in 2004 (Table 2).

**Table 2:** Inferred census data 1991–2006, Manu National Park, Peru.

	INFERRED							
Year	Census total	No. res. groups	No. trans.	No. litters	No. cubs	Mean res. group size	Mean litter size	Mean no. cubs/group
1991	48	7	7	3	8	5.9	2.7	1.1
1992	49	9	6	8	15	4.8	1.9	1.7
1993	44	8	2	4	9	5.3	2.3	1.1
1994	42	7	4	5	9	5.4	1.8	1.3
1995	43	8	4	3	7	4.9	2.3	0.9
1996	48	8	2	5	14	5.8	2.8	1.8
1999	64	10	4	6	14	6.0	2.3	1.4
2000	78	12	11	7	16	5.6	2.3	1.3
2001	81	12	13	7	13	5.7	1.9	1.1
2002	82	12	14	9	18	5.7	2.0	1.5
2003	82	11	14	8	14	6.2	1.8	1.3
2004	88	11	16	9	23	6.5	2.6	2.1
2005	87	12	19	7	11	5.7	1.6	0.9
2006	81	11	18	6	14	5.7	2.3	1.3
TOTAL	917	138	134	87	185	5.7	2.1	1.3

**Figure 4:**
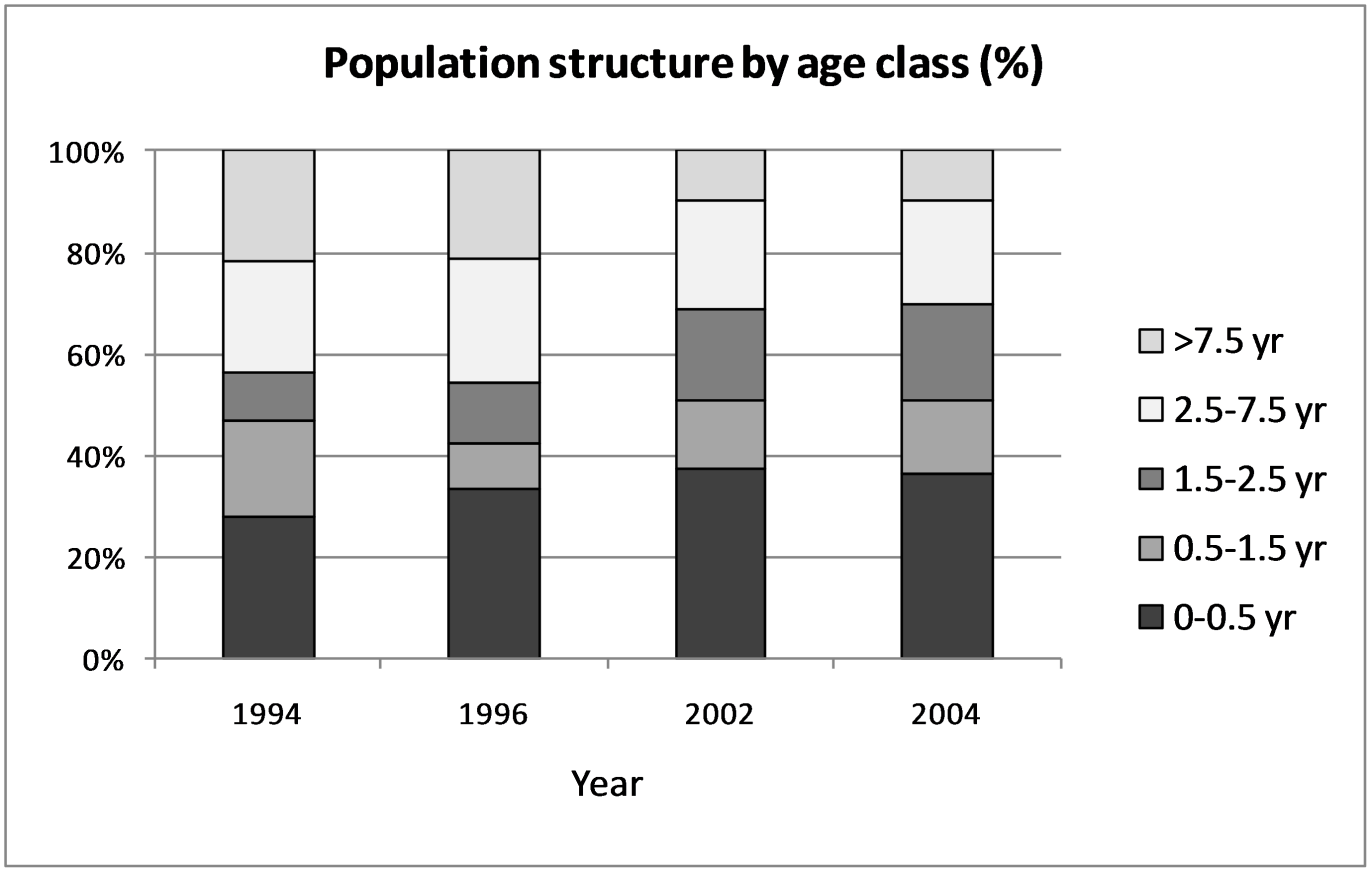
Population structure by age class, years 1994, 1996, 2002 and 2004.

Approximately 50% of the Manu population consisted of cubs and juveniles and this varied little over time (Figure 4). Overall, the population was comprised of younger individuals in the 2002 and 2004 censuses, compared to 1994 and 1996 censuses, which is consistent with the observed population growth.

The life table (Table 3, Table S1) generated values of 1.28 for the net reproductive rate (Ro), 8.21 yrs for the generation time (T), and 0.03 for the intrinsic rate of increase (r) of the population. This value of r implies a doubling time (derived from the equation for exponential growth Nt = No.e^rt^ by setting Nt/No = 2, and solving for t) of approximately 23 years, which is consistent with the overall trend observed in Figure 1.

**Table 3:** Cohort life table for the Manu giant otter population, 1991 to 2006 cohorts (excluding 1997 and 1998).

x (Age)	Nx	mx	Sx	lx	lxmx	xlxmx	Ex
0.5	177	0.00	0.63	1.00	0.000	0.000	3.49
1.5	111	0.00	0.80	0.63	0.000	0.000	3.97
2.5	89	0.00	0.71	0.50	0.000	0.000	3.71
3.5	63	0.05	0.63	0.36	0.017	0.059	3.83
4.5	40	0.63	0.78	0.23	0.141	0.636	4.45
5.5	31	0.97	0.81	0.18	0.169	0.932	4.45
6.5	25	1.04	0.92	0.14	0.147	0.955	4.28
7.5	23	1.17	0.74	0.13	0.153	1.144	3.57
8.5	17	1.76	0.82	0.10	0.169	1.441	3.47
9.5	14	2.00	0.64	0.08	0.158	1.503	3.00
10.5	9	2.11	0.78	0.05	0.107	1.127	3.11
11.5	7	2.43	0.86	0.04	0.096	1.105	2.71
12.5	6	1.67	0.50	0.03	0.056	0.706	2.00
13.5	3	2.33	0.67	0.02	0.040	0.534	2.00
14.5	2	1.00	0.50	0.01	0.011	0.164	1.50
15.5	1	2.00	0.00	0.01	0.011	0.175	1.00
16.5	0	0.00	0.00	0.00	0.000	0.000	0.00

x(Age) = age interval (starting at 0.5 yrs which is when cubs are first censused), Nx = number of individuals per age class, mx = fecundity (half number of offspring born to parent aged x), Sx = age specific probability of survival to following year, lx = probability of survival from birth to age x, lxmx = average number of offspring born to female at age x (age specific contribution to reproduction), xlxmx = mother’s age when each offspring was born, Ex = life expectancy (average lifespan remaining for an individual of age x).

### Reproduction

At the time of censuses, cubs were aged approx. 0.5 yrs old, well after emergence from the den, which occurs at about 2 months. We observed four groups moving litters of young cubs between dens, estimated at between two weeks and one month old. Of the 17 cubs involved, 12 were subsequently recorded during the annual census. If typical, this suggests a pre census cub mortality of at least 30%. Entire litters could have been missed from the census if all their members died earlier. Litter size estimates are thus minima.

We present data on 30 adult females (15 known-age, 15 estimated-age) and 31 adult males (10 known-age and 21 estimated-age) which reproduced successfully, i.e., producing at least one litter to age 0.5 years. The average reproductive lifespan for adult females was 5.4 years (n = 30, SE 0.5, range 1.0–11.0), starting at age 3.0 which was the earliest recorded age of reproduction. However, litters were produced for an average of only 3.2 years (SE 0.43, range 1.0–5.0). Each year, therefore, several dominant females failed to raise a litter to 0.5 years. Average age of first parturition was 4.4 (n = 14, SE 0.43, range 3.0–9.0). The average reproductive lifespan for adult males was 5.2 (n = 31, SE 0.49, range 1.0–13.0), with 3.0 also being the earliest age at which males fathered their first recorded litter. Litters were produced for an average of 3.1 years (SE 0.39, range 1.0–9.0), with the average age at first litter being 4.6 (range 3.0–6.0) (Table 4).

**Table 4:** Baseline demographic variables for giant otters in Manu National Park.

	Demographic variable	Average value (years)
**MALES**	Age at dispersal	2.9 (n = 19)
	Age at first recorded reproduction	4.8 (n = 10)
	Longevity	4.8 (n = 33)
**FEMALES**	Age at dispersal	2.5 (n = 11)
	Age at first recorded reproduction	4.4 (n = 14)
	Longevity	4.6 (n = 23)
**POPULATION**	Litter size at age 0.5 yrs.	2.1 (n = 87 litters)
	Litter size at dispersal (independence)	1.1 (n = 87 litters)

Reproductive animals usually remained in their home range until death or disappearance (in 38 of 40 cases). Immigrants were only recruited into a resident group if they claimed the dominant breeding status. It is not clear whether these changes of one member of the breeding pair involved active displacement by the newcomer or whether the newcomer simply occupied an already vacant position (i.e. following the predecessor’s death). Incest was never recorded.

Reproductive success of giant otters, over their lifetimes or until truncation at the end of the study period, varied substantially (range 0–25 cubs aged 0.5 years). Of 41 reproductively mature females, 11 (27%) were not recorded to produce a single litter, 17 (41%) produced only one or two litters, and 13 (32%) produced three litters or more. On average, breeding females produced 6.9 cubs per lifetime (se 1.17). Of 50 reproductively mature males, 19 (38%) were not recorded to produce a single litter, 16 (32%) produced only one or two litters, and 15 (30%) produced three litters or more. Breeding males produced a mean of 6.7 cubs per lifetime (SE 1.08). The longest reproductive lifespan was 13 years for males and 11 years for females. The variance in reproductive success thus tended to be higher in males compared to females.

#### Litter number, size and seasonality

In total, 172 cubs were observed in 78 litters between 1991 and 2006. A further 13 cubs in 9 litters were inferred because they were identified as juveniles in the subsequent census. Of the total of 185 cubs (Table 2), 177 were uniquely identified using their throat markings. As a norm, the dominant female of a giant otter group produced one litter per year. However, in 2001, the same female in Cocha Salvador produced two litters (1+5 cubs) in one year. Observed litter size at time of census ranged from 1 to 5 with a mode of 2 (post-emergence mean = 2.2, SE = 0.04, n = 78).

**Figure 5:**
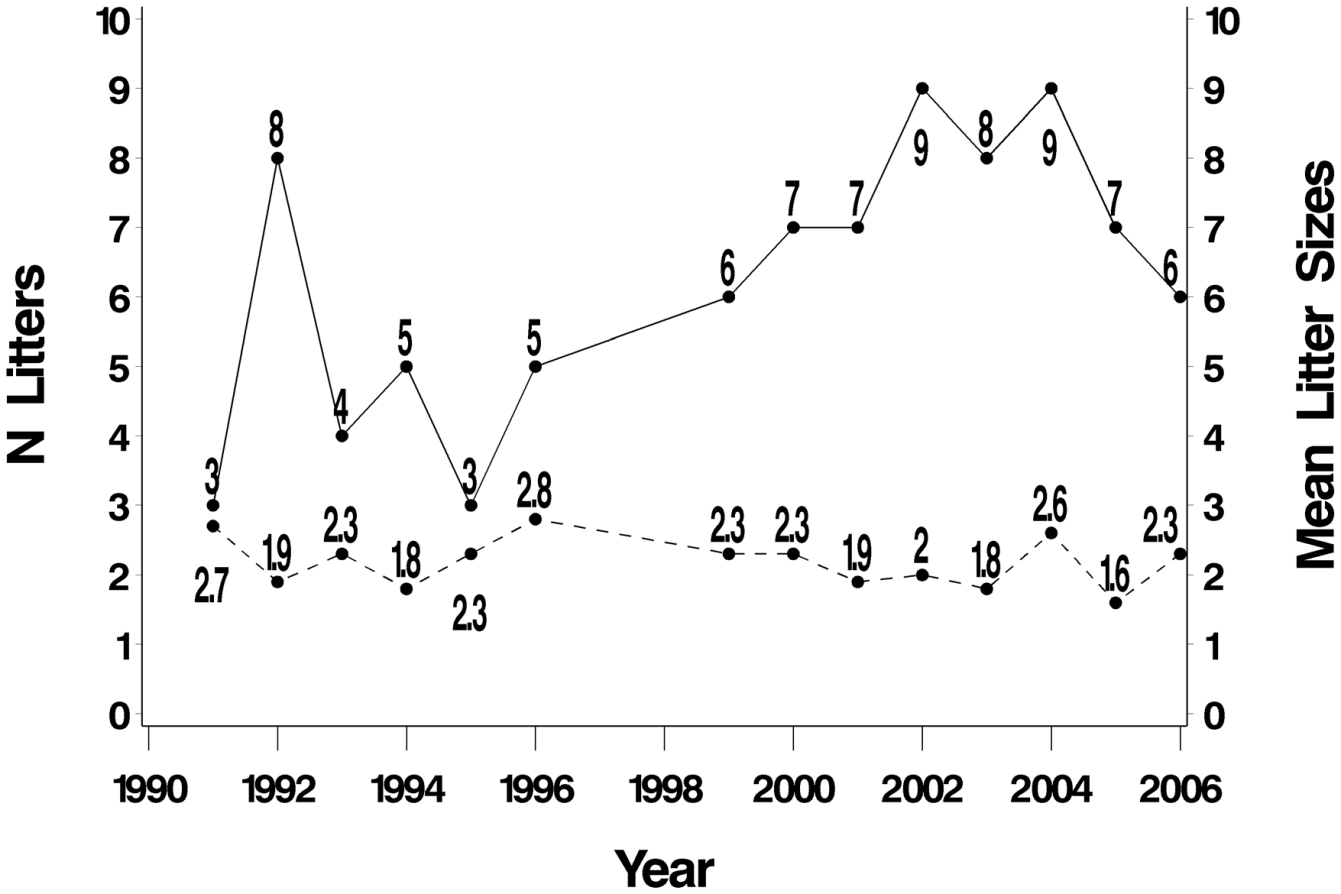
Trends in number of litters (solid line) and mean litter sizes (for those litters).

Litters were born in all four quarters of the year, but the number recorded varied between quarters, showing a high degree of seasonality. During the dry season (April through September), water levels are at their lowest, fish densities at their highest (resource concentration) and habitat conditions at their most stable. Over all years, 67.9% of litters were born in the second quarter (beginning of the dry season) and 20.8% in the third quarter (end of the dry season). Only 11.3% were born during the wet season (first and fourth quarters combined). The number of litters produced annually in the study area increased significantly over the study period (Pearson’s r = 0.63, P = 0.01) (Figure 5). The observed mean number of cubs born per resident group year (i.e. including years when a group was resident but no cubs emerged), was 1.5 (n = 117 group years). The size of litters when at least one cub emerged averaged 2.1 (SE = 0.10, n = 14), and there was no evidence for a temporal trend (Pearson’s r = −0.26, P = 0.36, n = 14).

#### Breeding Tenure

A reproductive individual’s tenure typically ended in disappearance. These individuals probably died, since they were never seen again in the study area. In two cases, however, dominant females were displaced by another female in the group (a sister and a daughter). Both displaced females stayed on in their groups until disappearance, one for a further two years. It is not clear why or how these displacements occurred. Once otters become reproductively dominant, individual differences in breeding success depend principally on the duration of dominance tenure (Table S2), although breeding route may also play a role. Routes to breeding are as follows: females can either inherit the dominant position in their natal group or they can form a new breeding group elsewhere. Males were never observed to become dominant breeders in their natal groups and either form a new group or immigrate into an existing group to occupy the reproductive male position. Becoming an extra-group breeder was not observed.

In all cases (n = 11) when vacancies arose for a reproductively dominant female, it was a subordinate female otter from the oldest cohort that inherited the position, though she was not necessarily the only female in that cohort. In none of these cases was the male partner related to the philopatric female. Where more than one subordinate female was present in the oldest cohort, we could not determine the factor(s) that influenced which of the siblings became dominant. The average age at which philopatric females were first recorded with a litter was 3.89 (n = 9), compared to an average age of 5.25 yrs for non-philopatric females (n = 4).

In 11 instances the breeding male disappeared and was replaced by an immigrant adult male. Cubs of the original pair were invariably adopted by the immigrant males and groups remained stable throughout the transitions, usually producing new litters the following year. In each case, it was not possible to determine whether the former male died due to natural causes, or was evicted from the group by the immigrant male.

### Survivorship, Longevity, and Status

**Figure 6:**
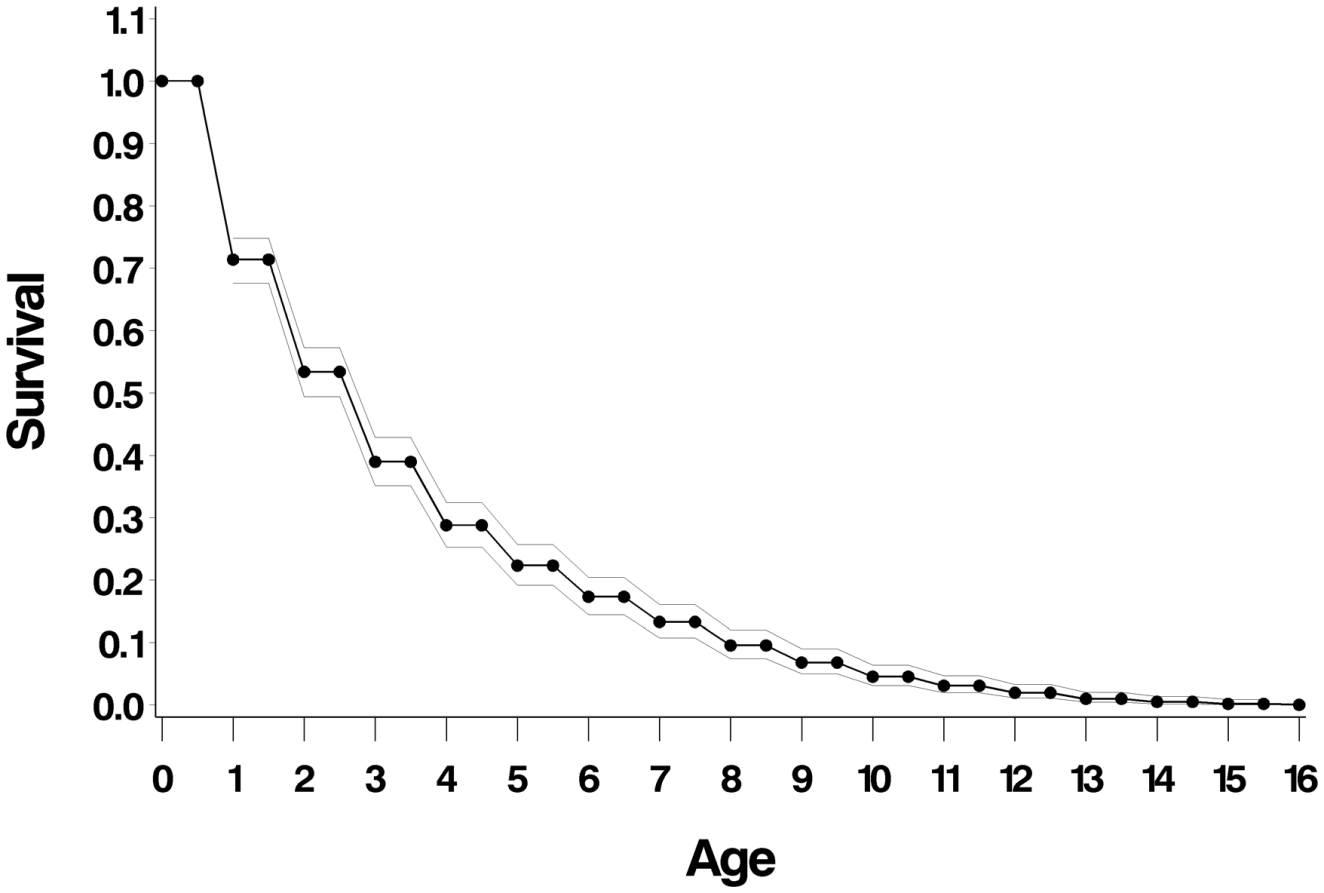
Survivorship from birth, all cohorts (n = 177).

The population survivorship curve (Figure 6) includes data from all cohorts censused (N otters = 177, Table S3). Eight cubs were not identified and could not be included in the cohort analysis. Mortality is highest for cubs and for dispersing age classes. Of 177 cubs, 111 (63%) survived to the next annual census. Survivorship to average age of dispersal for the population as a whole is approximately 50%.

**Figure 7:**
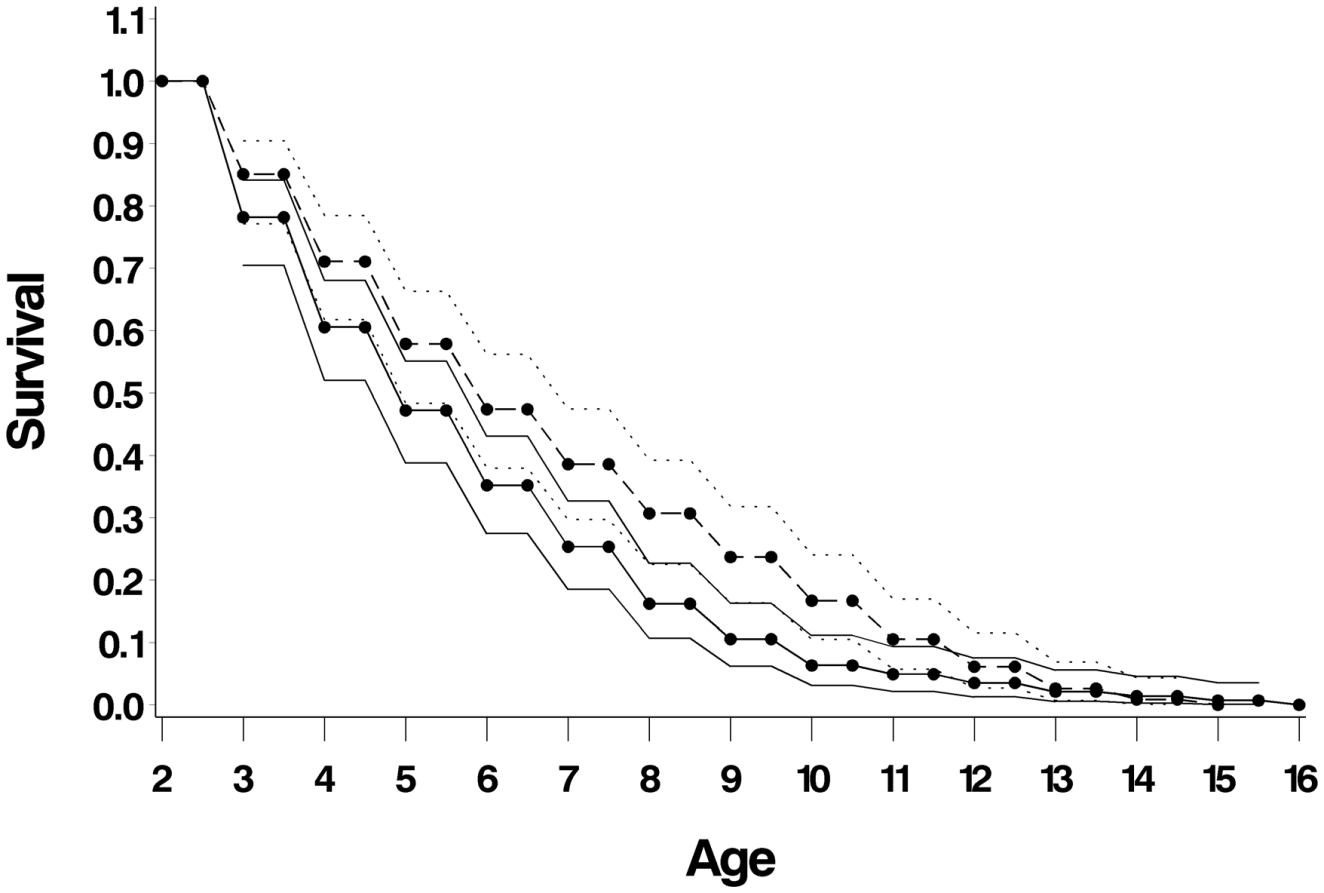
Post-independence survivorship of males (n = 31, solid line) and females (n = 17, broken line).

**Figure 8:**
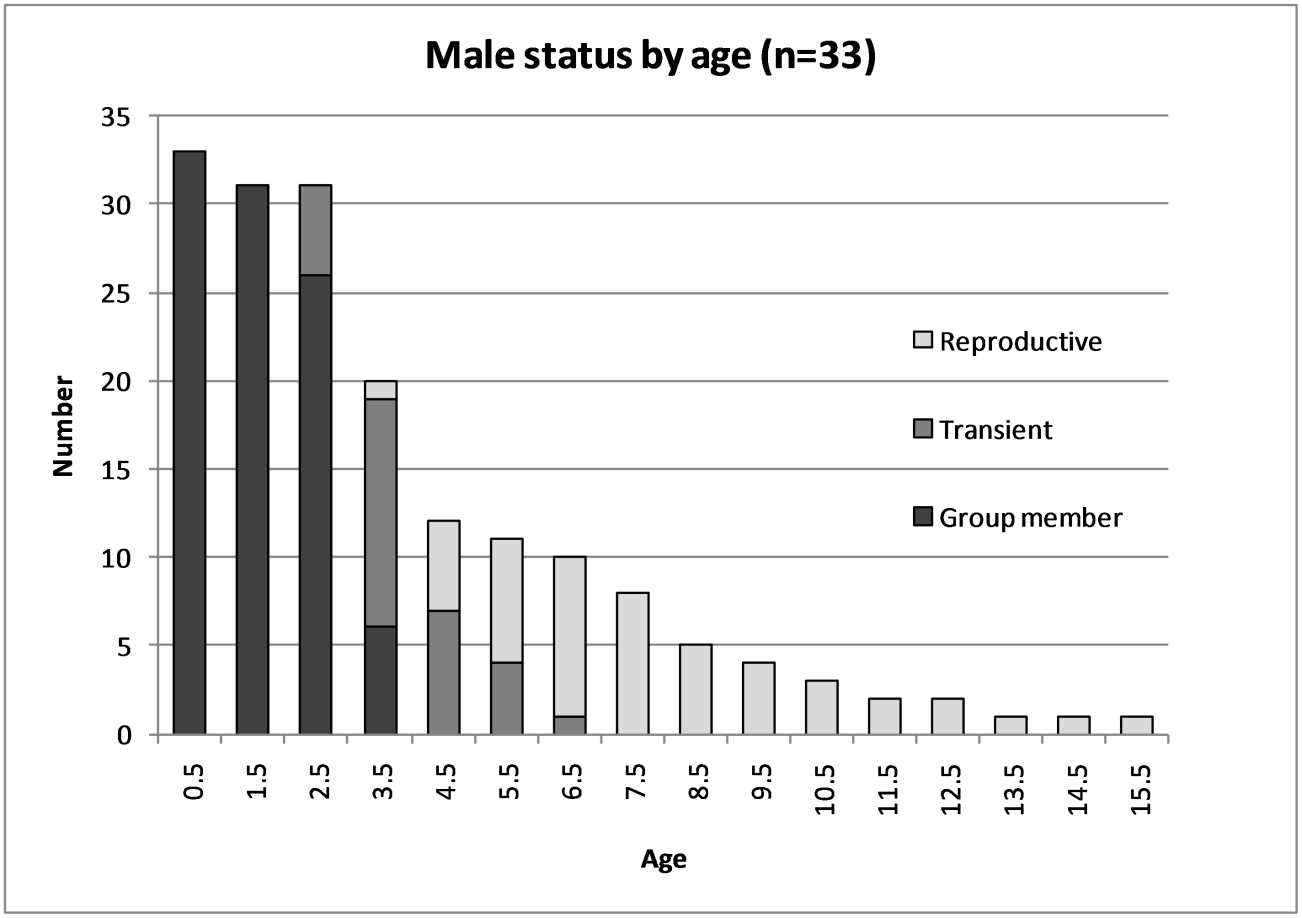
Male status (R - reproductive group member, T - transient, G - non-breeding group member) by age age class (n = 33).

**Figure 9:**
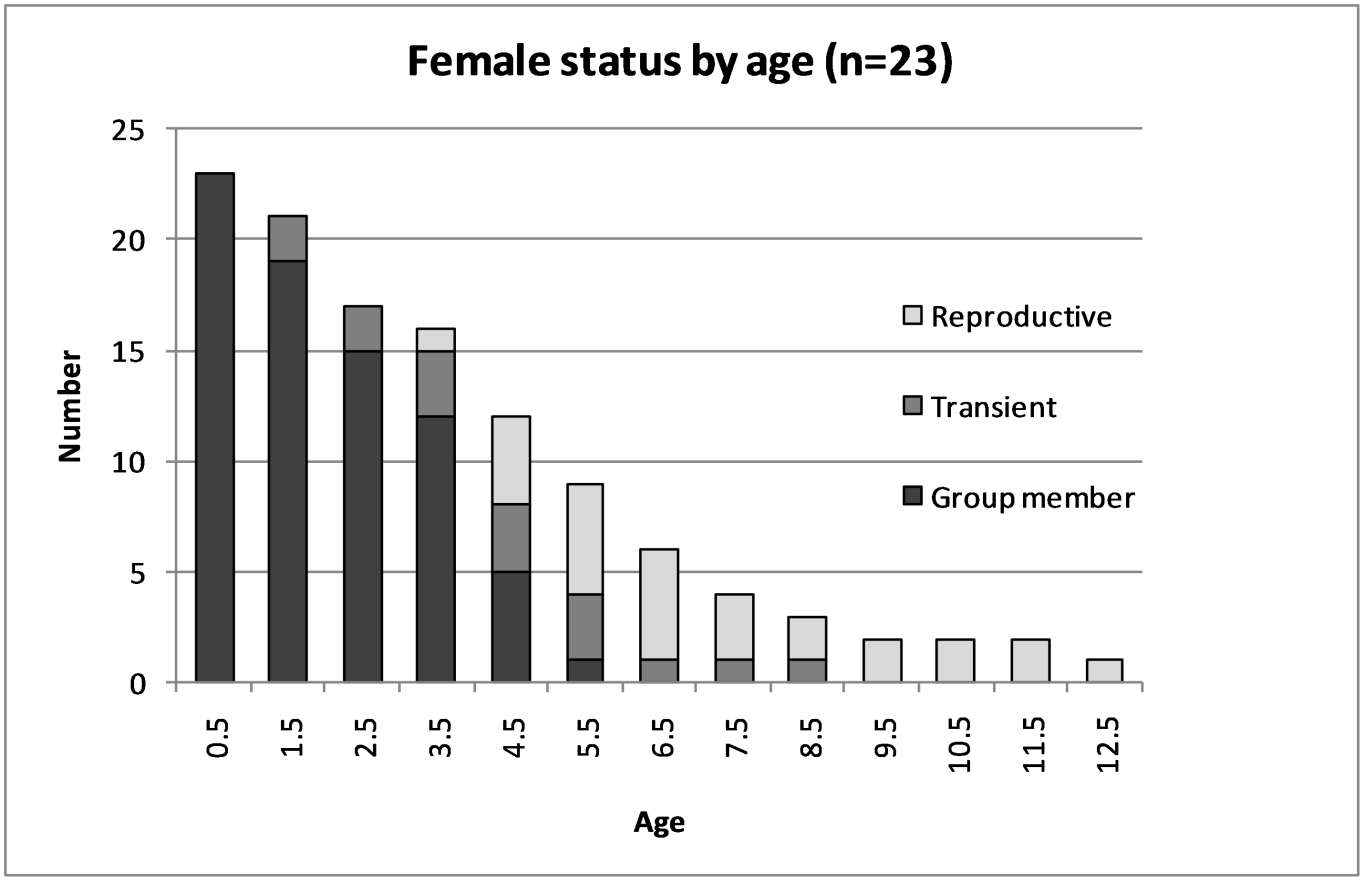
Female status (R - reproductive group member, T - transient, G - non-breeding group member) by age age class (n = 23).

Post-independence survivorship differed between the sexes (SAS PROC LIFESTEST log-rank χ^2^ = 5.9, P = 0.015) (Figure 7); this is associated with a marked pulse in male dispersal at age 3.0, resulting in lower male survivorship at this time. All males have left their natal groups by age 4.5. Females start dispersing as young as 1 year old but may stay on in their natal territories up to two years longer than males (6.5 years). After a period of high survival during the early adult years (4.5–7.5), survival decreases again, first slowly and then more rapidly in later years. Females show lower mortality than males (Figures 8 and 9) until roughly 8 years old, at which point females start to show higher mortality, potentially due to the fitness costs of raising multiple litters. Life expectancy peaks between 4.5 and 5.5 years, with the longest-lived male and female last seen at 15.5 years and at least 13.5 years old respectively. These are the longest recorded ages for free ranging giant otters.

## Discussion

### Population Trends

Giant otters are towards the slow end of the fast-slow continuum in mammal life history strategies explored by Promislow & Harvey [35]. It appears, retrospectively, that the population in the floodplain of Manu National Park was still in a process of recovery from the impact of the pelt trade - not all the available territories were occupied when this study was initiated in 1991. Over the next 16 years (1991–2006), the population grew (r = 0.03), with this being due to an increase in (1) the number of breeding groups (from 7 in 1991 to 12 in 2000), and therefore the number of litters per year, (2) the size of groups (from a mean of 4.8 in 1992 to 6.5 in 2004), and (3) the number of transients in the system (from 2 in 1993 to 19 in 2005). Since the environmental capacity has not increased (the area has been protected since 1973 when the Park was established, almost 20 years before this study was initiated), this suggests a return to carrying capacity after the hunting decades. As all suitable territories in Manu have now been occupied by resident groups and there are more non-breeding transients in the system, intraspecific competition is likely to increase in the future, with a consequent decrease in r.

### Individual Demographic Variables

Male and female giant otters show very similar traits with respect to average ages at first litter (female 4.4 yrs, male 4.6 yrs.), average reproductive life-spans (female 5.4 yrs., male 5.2 yrs.), and average cub productivity (female 6.9, male 6.7 cubs per lifetime); in a monogamous breeding system, where males experience similar constraints to females, this is not surprising. The high degree of reproductive skew found in giant otters, comparable to that in other highly cooperative breeders such as the dwarf mongoose (*Helogale purvula*) [36], the African wild dog (*Lycaon pictus*) [13], and the meerkat (*Suricatta suricata*) [37], means that, over all censuses, only 30% of the population consisted of breeding animals. Furthermore, of the giant otter reproductive pairs that produced litters, almost all did so only once per year, producing relatively small litters in those years when any cubs emerged (mean size = 2.1). By age of dispersal, approximately 50% of offspring have died. High dispersal mortality, especially among transient males, has also been documented in this study.

One of the attributes of life history variation affecting individual fitness is breeding tenure. In this study, dispersing females that are successful in forming new groups experience a delay in age at first reproduction, decreasing lifetime reproductive success significantly. Partner compatibility (i.e. of similar age) and territory quality also play important roles [unpublished]. When members of a breeding pair of a similar, young age occupy a high quality territory, they have the potential for high reproductive success: the five most productive breeding pairs (defined as producing 10 or more cubs during their shared breeding tenure) produced at least 10, 11, 19, 21 and 25 cubs within their respective territories.

Thus, the combination of (1) physiological traits such as late age at first reproduction and long generation time, (2) a high degree of reproductive skew, (3) small litters produced only once a year and a pre-census cub mortality of 30%, (4) a further 50% mortality up to age of dispersal as well as high mortality amongst dispersers (especially males), (5) plus the influence of breeding pair compatibility, all help to explain the low intrinsic rate of increase of the population, slowing its ability to recover from the pelt trade in the past or possible future disease outbreaks. Furthermore, in Manu National Park, lakes comprise a crucial and patchily distributed resource-rich habitat within a giant otter territory. The area of lake encompassed by a territory predicts group structure: both the size of the groups and the fecundity of the breeding female are proportional to the total lake area within the group territory [unpublished]. Hence, extrinsic factors such as territory quality and distribution may also limit r and explain why protected giant otter populations in Manu and elsewhere have taken decades to recover from the crash induced by over-hunting.

### Implications for conservation

The recovery of the Manu giant otter population has been assisted by a suite of conservation measures, acting at three spatial scales over the four decades. First, a policy measure at the continental level was the 1975 listing of the giant otter in Appendix 1 of CITES. This was preceded by national legislation banning commercial hunting in Peru in 1973, coupled with a worldwide decline in the demand for wild pelts. Next came the creation and implementation of large protected areas that contained remnant giant otter populations, including Manu. These areas, resulting from conservation and land use planning decisions at the national level, have been crucial in stabilizing populations. Finally, regional and local habitat management measures such as Protected Area zoning and oxbow lake management plans have been implemented in Madre de Dios since 1990 [18]. These have focused on reducing human pressure on key habitats of the species, with the objective of maintaining habitat quality and maximizing giant otter reproductive success.

In parallel with the recovery of some giant otter populations, however, protected areas such as Manu and other giant otter habitats of the Madre de Dios watershed have come under increasing pressure. As one of the few remaining colonization frontiers of the Amazon, the Department of Madre de Dios has seen considerable mining, logging and agricultural expansion over the last two decades. The average rate of human population growth between 2002 and 2012 was 3%, the highest in the country [38]. It is estimated that 40% of this population consists of recent immigrants attracted by land availability and job opportunities [38]. Furthermore, in the period 2002–2011, the average annual growth of the economy in Madre de Dios was 7.3%, higher than the national average growth of 6.4% [39].

The most important economic activity by far is alluvial gold mining, more than half of it informal and illegal. Driven by increasing gold prices and new road access, the extent of gold mining in the Madre de Dios region increased from less than 10,000 hectares in 1999 to more than 50,000 hectares in 2012. The rate of expansion jumped from 2,166 ha per year before 2008 to 6,145 ha per year thereafter. The Department of Madre de Dios generates 70% of Peru’s artisanal gold production and Peru’s mercury imports have increased exponentially (∼175 t in 2009), 95% of which is used in artisanal gold mining, resulting in the release of large quantities into the atmosphere, sediments and watersheds [40]. In a Manu study analyzing mercury and methyl mercury levels in fish muscles, Gutleb et al. [41] found that total mercury levels in 68% of fish muscles exceeded the tolerable level for the European otter (*Lutra lutra*) and 17.6% exceeded 0.5 mg kg^−1^ fresh weight, the common standard for human consumption.

The aquatic systems, their floodplains, and forests associated with these, are thus amongst the most threatened habitats in Madre de Dios, both due to their natural limited extent, as well as the concentration of human activities such as mining and agriculture in these areas [42]. Thirty percent of riverine forests in Madre de Dios (this figure includes those in Protected Areas) have already been destroyed. If Protected Areas are excluded from the analysis, the situation is much worse.

Giant otter abundance in Manu is characterized by relatively high local densities (5–10 individuals per square kilometre of lake) and low absolute densities (less than 1 individual per 100 square kilometres of rainforest), due to the limited total surface area of suitable aquatic habitats (particularly lakes) and their patchy distribution over the landscape. The annual giant otter censuses (recording a maximum of 88 inds.) have allowed us to estimate the total giant otter population in Manu National Park (including the headwaters) to be between 100 and 130 animals, that is, fewer than 22 groups or breeding pairs (given an average group size of six animals).

Other studies [18], [33] suggest that the Madre de Dios River itself, twice as wide as the Manu River, provides the highest quality habitat for the species; its large oxbow lakes and wetlands could support large giant otter groups with high reproductive output and, potentially, the largest sub-population of the watershed. The Madre de Dios floodplain is also a natural corridor through which giant otters of the Manu, Los Amigos, Heath and other tributaries could disperse. As no single protected area in the Madre de Dios region is close to harbouring a demographically viable population (N_e_ ≥50), interchange of individual otters between the sub-populations is necessary if we are to lower the probability of immediate risk of local extinction in the face of future threats.

We recommend that an aquatic habitat conservation corridor be consolidated along the Madre de Dios River, ensuring the restoration of otter habitats impacted by mining and deforestation therein. Only by facilitating the return of giant otters in the Madre de Dios floodplain will the population of the entire watershed reach N_e_ ≥500 individuals, our minimum conservation goal for a genetically viable population [43]. Effective Protected Area design and broader wetland landscape management initiatives are therefore critical for the long term conservation of the species in Madre de Dios.

## Methods

### Census protocol

The majority of the data analysed here were collected during 14 annual dry season censuses in Manu National Park between 1991 and 2006 (excepting 1997 and 1998). Censuses were coordinated by Staib and Schenck between 1991 and 1996, by Groenendijk and Hajek between 1999 and 2005, and by Calvimontes in 2006. Successors were trained in census methodology in the field by the preceding census team, and the same field equipment was used. Additional specific otter observations, film footage and photographic evidence collected between 1987 and 1991 were contributed by Andre Baertschi, and between 2006 and 2009 by Lisa Davenport and the Frankfurt Zoological Society; these 73 extra annual data points allowed us to complete a number of individual giant otter life histories.

The primary objective of the censuses was to count, identify, and record the status of all giant otters within the floodplain of the Manu River in order to determine population size and collect demographic data. Each census covered 230 river kilometres and a core group of 20 oxbow lakes (ranging in total surface area from 10.5 to 101.9 hectares, with an average of 36.6 hectares), together with 11 additional lakes which were surveyed less intensively; the census area (number of oxbow lakes and river kilometres) was constant between the three consecutive census teams. The censuses were completed within an average of 39 field days (range 32–42 days) at the end of the dry season, when giant otter litters of that year had emerged from the den (reducing the probability of not locating litters and of the occurrence of post-census births). The Manu River was navigated with a 15 m motorised canoe, while oxbow lakes were surveyed with an inflatable boat, following a population census protocol developed by the IUCN/SSC Otter Specialist Group [44].

In Manu National Park, Schenck [33] and Staib [16] found that home ranges of giant otters typically include one or more oxbow lakes, the associated stretch of river, and adjacent swamp areas and streams (which are inaccessible for observers and therefore make it impossible to define the exact extent of home ranges). One or two oxbow lakes form the core area or territory within each home range, maintained through defense and/or scent marking activities, and which hold resources critical to ensure successful reproduction. Rivers and streams are used as connection routes between the lakes within the home range. Otter groups tend to maintain these core territories throughout the year and from year to year; long-term spatial fidelity therefore permits retrospective inference of a group’s presence even when group composition changes (for example, through inheritance). Territories do not appear to change in size with the seasons, although different microhabitats are used in accordance with fluctuations in water levels. During the high waters of the rainy season, the otters spend more time in *Mauritia* palm swamps, streams, and flooded forest areas surrounding the lakes, while during the dry season activity is mainly concentrated on the oxbow lakes themselves. Overlapping of neighbouring home ranges was not observed in Manu, but cannot be discounted.

In areas of low human disturbance, such as Manu National Park, the characteristic behaviour of giant otter groups to investigate intruders ensures that the surveyor is usually not avoided, even by non-habituated groups (although transients are shy and more elusive). Most otter groups reacted to the survey canoe by approaching and repeatedly craning head and neck straight out of the water, a behaviour known as ‘periscoping’. Each individual is identifiable from infancy by its unique pale throat marking [4], making it possible to avoid double counting and to follow the life histories of animals over successive years. Rarely, an animal lacked a throat marking (n = 3 out of 294 inds.).

Under field conditions, body size is not a reliable indicator of gender or age; some males were noticeably smaller than their partners and older animals were not always the largest [16]. In order to identify individuals and classify them into age categories, we combined field observation of behaviour to establish status (whether cub, juvenile, sub adult, adult, or member of breeding pair), with subsequent review of video footage to establish ID and gender. Although cubs and juveniles are easily recognized by their size and behaviour (e.g. begging), sub adults and adults are more difficult to distinguish from each other. Sub adults and adults in the different resident groups were determined as such with subsequent censuses, as older animals dispersed and cubs identified in previous censuses matured.

In order to determine gender differences in demographic variables, otters were sexed. Sexes were distinguishable when individuals were entirely out of the water, usually when basking or grooming on logs. Until 2002, all sexing was based on such opportunistic sightings, so animals that were longer in the population were more likely to be sexed. Sexing was carried out by observing teats in adult, parous females (four permanently elongated teats due to lactation), or testicles in males: under field conditions, the male’s scrotum does not become clearly evident until he is at least one year old [16]. The breeding male could be identified by its behaviour, specifically that of intensive marking, and by its year-on-year permanence in the group (non-breeding males do not stay). However, sexing was more difficult in adult females that had not lactated, or in cubs, juveniles and sub adults of both sexes. As a consequence, many individuals, particularly transients, could not be sexed until 2002, when we discovered a simple and effective method of sexing giant otters, regardless of age. When they visit latrines, otters often defecate and urinate simultaneously [4] (fecaluria). Males can then be distinguished from females by the larger space between the sources of the urine and scat streams [45].

All field research was conducted in accordance with the requisite permits awarded by the Instituto Nacional de Recursos Naturales (INRENA), later Servicio Nacional de Areas Naturales Protegidas por el Estado (SERNANP). No invasive sampling methods were used and no giant otters were captured.

### Data treatment

Ages were categorised as: ‘cubs’ up to 0.5 yr, ‘juveniles’ between 0.5 and 1.5 yr of age, ‘sub adults’ between 1.5 and 2.5 yr old, and ‘adults’ at and over 2.5 yr of age. Animals were censused towards the end of each age class, i.e., cubs were first censused close to 0.5 years old, after emergence from the den.

Each sighting was recorded in a throat pattern catalogue, along with the location, date, status as interpreted from observation of behaviour (cub, non-breeding group member, transient, or breeding member) and sex, if determined. Each individual otter was noted as either resident (if already present in the study area in 1991 or 1999, or if born in the study area between 1991 and 2006) or immigrant, and labelled for each data point as positively identified, ID uncertain, or inferred.

Inferred animals or groups are those which are assumed to have been in the population and study area in a given year when they were identified in both the previous and subsequent census, and were included in the analyses; in the case of groups for which there was a gap in data continuity, the number of individuals was estimated to be the average of group sizes in the years before and after that gap (provided key group members could be identified). Animals that were seen in 1996 and again in 1999 were inferred for the intermediate years. Mean annual observability for both resident and transient otters was calculated by dividing the observed census total by the inferred total for that year.

The Manu population was sub-divided into known-age (i.e. first recorded as cubs in the study area) and estimated-age otters (first recorded as transients or as group members). Data from known-age individuals were used to assign ages to the estimated-age animals.

In order to develop survivorship and fecundity schedules and a population life table, we carried out a cohort analysis [46] with 177 known-age individuals whose life histories were known from birth to disappearance. There are many similarities between the social organizations and behaviour of the giant otter and the African wild dog (*Lycaon pictus*): both live in permanent packs or groups, only the dominant female is assured of breeding, reproduction is monopolized by the dominant male, and subordinates of both sexes help to raise young [47]. We therefore followed a similar approach to that adopted by Creel and Creel [13] for wild dogs. Both their study in Selous and this study in Manu relied on determining age- and sex-specific annual rates of survival rather than mortality: no dead giant otters were encountered during the study hence natural causes of mortality are uncertain.

If giant otters seen in an earlier census were not observed in subsequent surveys, they were considered dead. Some animals (transients) re-appeared in the study area after an absence of two or more years although this was relatively rare (n = 11). As no surveys were conducted in 1997 and 1998, animals that were last seen in 1996 were assumed to have died in 1997.

We adjusted apparent survival to allow for undetected emigration of transients and groups on the assumption that this was equal to the observed rate of immigration by unknown transients and groups [13] (i.e. the Manu floodplain is assumed to be neither a source nor sink population within the wider Upper Madre de Dios river ecosystem). Since paternity is thought to be known and only the dominant pair in the group breeds, we divided cub production equally between the sexes and calculated ‘mx’ as half the total number of offspring for each parent. We used both male and female offspring because many cubs could not be sexed.

We estimated the ages of 30 transient and reproductive immigrants to the Manu system using our known-age population and distributed these individuals amongst the cohort and fecundity age classes as accurately as possible according to their sex (male, female, unknown); this has the consequence of extending the life-spans of some dispersers and breeding animals, rather than assuming they all died on disappearance. Separate survivorship curves for males and females upon reaching sexual maturity (2.5 yrs) were constructed where the sex was known (males n = 31; females n = 17, from 177 individuals).

From the life table (Table 3) we calculated the net reproductive rate Ro (∑l_x_m_x_), generation time T (∑xl_x_m_x_/Ro) and the intrinsic rate of increase r (ln (Ro)/T) for the Manu population, following Krebs [46] and Creel and Creel [13]. The data analysis for this paper was generated using [SAS/STAT] software, Version 9.12 of the SAS System for Windows 7, copyright 2002–2008.

## Supporting Information

Table S1 **Cohort analysis and life table.**
(XLS)

Table S2 **Breeding pair duration and reproductive success.**
(XLS)

Table S3 **Survivorship.**
(XLS)
